# ATTITUDE - Addressing attrition in longitudinal cancer cohorts: an in-depth qualitative analysis of experiences and perspectives on participation in longitudinal studies among breast cancer survivors

**DOI:** 10.1007/s10549-026-07904-w

**Published:** 2026-02-11

**Authors:** Elise Martin, Mariam Chichua, Camila Kelly Chiodi, Petya Zyumbileva, Pietro Lapidari, Martina Pagliuca, Emma Gillanders, Aude Barbier, Aurélie Bertaut, Diane Boinon, Anne-Laure Martin, Sibille Everhard, Liliane Golli, Christelle Jouannaud, Courèche Kaderbhai, Benoîte Mery, Olivier Rigal, Maria Alice Franzoi, Ines Vaz-Luis, Antonio Di Meglio

**Affiliations:** 1 Université Paris-Saclay, Gustave Roussy, Inserm, CESP, F-94805 Villejuif, France; 2 Gustave Roussy, 114 Rue Edouard Vaillant, 94800 Villejuif, France; 3 University of Milan, Milan, Italy; 4 European Insitute of Oncology IRCCS, Milan, Italy; 5 Scuola Superiore Meridionale, Naples, Italy; 6 Centre Georges-François Leclerc, Dijon, France; 7 Department for the Organization of Patient Pathways (DIOPP), Gustave Roussy, Villejuif, France; 8 Laboratoire de Psychopathologie Et Processus de Santé, Université Paris Cité, F-92100 Boulogne-Billancourt, France; 9 UNICANCER, Paris, France; 10 Institut Godinot, Reims, France; 11 Centre Léon Bérard, Lyon, France; 12 Centre Henri Becquerel, Rouen, France

**Keywords:** Longitudinal cohort studies, Attrition, Patient experience, Retention, Breast cancer

## Abstract

**Purpose:**

Longitudinal studies are essential for investigating outcome evolution among cancer survivors. However, longitudinal designs pose specific challenges, such as attrition and premature dropout. We aimed to assess experiences and perspectives on participation in longitudinal studies among survivors of early-stage breast cancer, and inform interventions aiming to reduce attrition.

**Methods:**

Motivation, facilitators, challenges, and perspectives regarding participation in a longitudinal study were qualitatively assessed via interviews and focus groups. A thematic content analysis was performed.

**Results:**

Between May and August 2023, 30 patients previously enrolled in the longitudinal CANTO cohort study (NCT01993498) were included: 17 participated in individual semi-structured interviews and 13 in two separate focus groups involving distinct participants. We identified four key themes: (1) joining the study as an expression of purpose and personal security, (2) ongoing engagement, as a response to flexible processes, a reassuring study environment, and personal commitment, (3) ongoing engagement, challenged by accumulating (practical and emotional) burdens and a reduced sense of connection to the study, and (4) participant-identified needs for a more supportive study experience. Findings emphasized the importance of enhancing communication with study participants, flexibility enabling decentralized participation, reducing burden of patient-generated data via repetitive questionnaires, supporting navigation of study procedures and minimizing the risk of a digital divide, and routinely disseminating study findings.

**Conclusion:**

These findings will inform strategies to reduce attrition and enhance the overall participant experience in longitudinal studies.

**Supplementary Information:**

The online version of this article (10.1007/s10549-026-07904-w) contains supplementary material, which is available to authorized users.

## Introduction

Longitudinal study designs have been used to follow cancer patients and survivors throughout their disease trajectory. In these studies, participants are assessed at multiple time points for the outcomes of interest, such as quality of life, psychological and social factors, or long-term side effects [1–4]. While this design may lead to an in-depth and rich dataset, it also has several challenges, including frequent dropout and missing data. Indeed, a longitudinal design may pose a challenge to ensure that complete data will be collected from each participant at all time points [5]. On the one hand, the issue of missing data points has been addressed by developing analytic tools and methods capable of handling such data [6]. On the other hand, the focus has progressively shifted toward reducing attrition and improving data completeness. Attrition is defined as the *"reduction in the number of participants in a study as it progresses."*[7] Attrition may hinder the statistical power and representativeness of the study and lead to bias. Therefore, attrition-reduction strategies such as barrier-reduction, community-building, and tracking individual participants may be implemented in a study, however they often have a variable and inconsistent impact on retention outcomes [8].

In the context of oncology, attrition in longitudinal studies has been studied in patient populations with specific types of cancer, such as head and neck cancer and prostate cancer [9, 10], or with cancer patients dealing with particular conditions, such as cancer anorexia [11]. In the cancer survivorship field, one of the largest scale examples of a longitudinal cohort study is the Dutch registry, which contains data from over 30,000 survivors of various cancer types [12]. Data from this study show that attrition is more likely in participants who are older, less educated, experiencing depressive symptoms, and reporting lower health-related quality of life [13].

The ATTITUDE project (“ATTrition in longITudinal stUDies of cancEr survivors”) was designed to use both quantitative [14, 15] and qualitative methods to study attrition in longitudinal cohort studies and to inform interventions designed to enhance the overall patient experience. In the present manuscript, we present an in-depth qualitative analysis of breast cancer survivors’ experiences and perspectives on their participation in a longitudinal study, aiming to capture elements of motivation to enroll, the facilitators and challenges to sustained engagement, and suggestions for improvement.

## Patients and methods

### Participants

Participants were recruited in six comprehensive cancer centers in France (Gustave Roussy, Centre Henri Becquerel, Institut Godinot, Centre François Baclesse, Centre Georges-François Leclerc, and Centre Léon Bérard), among survivors of stage I-III breast cancer participating in the longitudinal CANTO cohort (NCT01993498). In the CANTO study, patients are assessed at diagnosis of breast cancer (a baseline visit is scheduled prior to initiation of treatment) and at follow-up visits at 1, 2, 4, and 6 years post-diagnosis. At each follow-up, participants undergo comprehensive assessments including clinical evaluations (conducted by a study nurse and covering system review, health behaviors, and treatments), socio-economic questionnaires (assessing education, income, and related factors), and patient-reported outcome measures (PROMs). Key PROMs administered include the European Organization for the Research and Treatment of Cancer (EORTC) Core Quality of Life Questionnaire and its breast cancer-specific module (QLQ-C30 and BR23, respectively), the EORTC cancer-related fatigue module (QLQ-FA12), the Hospital Anxiety and Depression Scale (HADS), and the Global Physical Activity Questionnaire (GPAQ-16). Completion time of such instruments ranges approximately from 45 to 60 min. Detailed descriptions of the study design and methodology have been published previously [16].

Recruitment for the present qualitative study used two approaches. (1) Centralized email invitations were sent to cohort participants, outlining the study and providing the research team’s contact details so that interested participants could reach out and indicate their willingness to be recontacted. Three waves of approximately 200 email invitations each were sent to reach a sufficient number of potential participants. Several emails failed due to invalid or inactive addresses, and most recipients completed only the accompanying quantitative survey [15]. (2) In addition, some invitations were conveyed via treating oncologists during follow-up visits. The most common reasons for declining participation were transferring care to another hospital, the long interval since diagnosis, and lack of interest.

Purposive sampling was employed to select participants with the aim of maximizing variation across several characteristics, including age, socioeconomic status (i.e., education, employment, marital status), type of residential area, and geographical distance from the treating center. This strategy was chosen to capture a diverse range of perspectives.

Enrollment and scheduling were managed exclusively by the research team. Interested individuals were recontacted and scheduled for participation, which took place using a virtual meeting platform. Participants were provided with a detailed, step-by-step guide on how to access and use the videoconferencing software via computer or telephone. Additionally, participants were offered an optional preliminary practice session to test connectivity and familiarize themselves with audio and video functions before the meeting. The researcher who conducted the interviews and facilitated the focus groups (EM) provided a phone introduction to each participant, during which she explained her background, research interests, and the purpose of the study.

We performed individual interviews to gain in-depth insight into personal experiences and sensitive issues less likely to be shared in groups. In addition, focus groups captured interactional dynamics and shared perspectives.

Based on a systematic review of empirical tests [17] and complementary empirical studies of saturation in interview and focus group research [18–21], we aimed to recruit 20–30 participants, which is consistent with achieving adequate thematic coverage in focused qualitative research with relatively homogeneous populations and well-defined objectives [17, 19]. Saturation was assessed iteratively during data collection and analysis, and recruitment ceased when further data did not generate additional analytic insights or meaningfully refine the themes being developed. This sample size allowed for capturing diverse participant experiences while ensuring analytical depth and study feasibility.

Before enrollment, all participants signed a written informed consent form. They were informed that they had the right to withdraw from the qualitative study at any time, however none of the participants withdrew. This study was performed in line with the principles of the Declaration of Helsinki. The study protocol was approved by the Gustave Roussy Institutional Committee Board (N°CSET: 3527).

### Procedures and data collection

The Consolidated Criteria for Reporting Qualitative Studies (COREQ), a checklist of 32 items, [22] was used to facilitate a comprehensive and thorough reporting (Appendix I).

A multidisciplinary team of medical oncologists, psychologists, social science and humanities researchers, and patient representatives, including from the Seintinelles participatory research network [23, 24] co-developed guides for interviews and focus groups that were implemented in the study, along with a short questionnaire to collect participant demographics (Appendix II). After briefly introducing the study and its aims, the researcher (EM, PhD, a female sociologist experienced in qualitative research) conducted the interviews and moderated the discussions during focus groups using the prompts included in the guides. The sociologist (interviewer/moderator) and a research team member (CK, MD, observer) were present during interviews and focus groups. They had no established relationship with participants prior to the study. Each participant took part in a single interview or focus group. The interviews and focus groups lasted approximately 20–50 and 80 min, respectively. Participants were encouraged to express experiences and perspectives regarding their involvement in a longitudinal study. The researcher guided the discussions to explore motivations, facilitators, challenges, and suggestions for improvement. Any spontaneous feedback that emerged from participant interactions, as appropriate, was also welcomed. In some instances, we explicitly invited participants to reflect on what they thought might be challenging not for themselves, but for other participants in longitudinal studies. Short field notes documenting contextual information and initial analytic impressions were taken during and directly after each interview and focus group.

All interviews and focus groups were audio recorded and transcribed verbatim by two transcribers (JA and EM), and the data was anonymized ensuring that participants’ personal information was not directly connected to the recordings. The transcripts were not returned to participants for comment and/or correction, nevertheless to reduces the bias of a single researcher and strengthen objectivity we did a researcher/analyst triangulation with two researchers (EM, MC) coding and analyzing the data.

### Analysis

A reflexive thematic analysis was conducted in 6 steps, consistent with a Big Q qualitative orientation that views meaning as situated and recognizes the researchers’ active role in theme development [26]:

1. Familiarization with the data: This step included reading and re-reading the transcripts of the audio recordings and researcher's field notes, organizing all the materials, and preparing them to be stored in the software for later analysis. Reflective thoughts were documented throughout to capture initial analytic observations and potential patterns of meaning relevant to the research question.
2. Coding: At this stage, all transcripts were coded line by line by two researchers (EM, PhD, sociologist; MC, PhD, psychologist) independently, using the NVivo 14 software to organize, store, and compare segments of text. A coding tree was created (Appendix III). This allowed for a more specific focus on the characteristics of the data. The coders refined the coding framework through an iterative process that included regular analytic discussions on comparing interpretations, refining codes, and supporting reflexive engagement with the data.
3. Generating initial themes: Themes were generated using a hybrid deductive–inductive approach. Following the aims of the study, key overarching themes were generated deductively, while the sub-themes were generated inductively by clustering similar codes of raw data. [27, 28]
4. Developing and reviewing themes: The themes were reviewed in accordance with Braun and Clarke’s guidelines [26] and modified where necessary to enhance coherence, distinctiveness, and analytic fit, ensuring that each theme captured a clear and meaningful pattern relevant to the research question. Following theme development, themes and sub-themes were mapped onto relevant Theoretical Domains Framework (TDF) (e.g., Knowledge; Beliefs about Consequences; Environmental Context and Resources; Social Influences; Emotion; Memory, Attention and Decision Processes; Reinforcement). An additional researcher contributed to this step (PZ). This analytic mapping enabled interpretation of each theme in relation to underlying behavioral determinants relevant to sustained participation in longitudinal studies [27]. The TDF consolidates key concepts from various behavior change theories, facilitating an analysis of the cognitive, emotional, social, and environmental factors influencing participants' sustained engagement. Continued participation is viewed as a complex behavioral process impacted by survivors' perceptions of the study's value, available support, emotional responses to repeated assessments, and contextual conditions. Interpreting themes through the TDF helps identify significant behavioral determinants related to attrition and retention, providing a foundation for developing strategies to enhance participant experiences and promote ongoing involvement in cancer cohort studies [28].
5. Refining, defining, and naming themes: Three researchers (EM, MC, PZ) refined and named themes, articulating the narrative each theme conveyed.
6. Writing up: The contents of each theme were described and illustrative quotations were selected. The final report was discussed with the broader multidisciplinary team. Participants were not asked to provide feedback on the findings (“participant checking”) to avoid increasing participant burden.

There was clear consistency between the data and the findings, as the themes were directly grounded in participants’ narratives and supported by the illustrative quotations presented in the Results section.

## Results

Overall, 17 patients were interviewed individually and two focus groups were conducted with eight and five participants, respectively (overall participant n = 30). The study included female patients with breast cancer that were 61 years old on average (ranging from 41 to 75). Detailed information about the participants’ characteristics is provided in Table 1.

**Table 1:** Participants’ characteristics

Characteristic, *n*	Total (*n* = 30)
Age in years, mean (Min–Max)	61 (41–75)
40–49	2
50–59	12
≥ 60	16
Highest education level	
Secondary school	1
High school	5
Professional education	3
< 5 years after high school (e.g., Bachelor degree)	7
5 years after high school (e.g., Master degree)	9
> 5 years after high school	4
Missing	1
Occupational categories	
Working	14
Retired	12
Unemployed	2
On disability	2
Family situation	
Single	3
In a relationship	6
Married	19
Divorced	1
Widowed	1
Type of town	
Village (< 2000 inhabitants)	5
Town (< 20,000 inhabitants)	9
Town (> 20,000 inhabitants)	16
Distance from the cancer center	
Less than 50 km	19
Between 50 and 150 km	10
More than 150 km	1

In the following section, we present main themes and sub-themes generated during this qualitative analysis, categorized under four structural categories: (1) motivation to participate, (2) facilitators of sustained participation, (3) challenges to sustained participation, and (4) suggestions for future improvement. Aligned with these categories, the respective four main themes presented here are: (1) joining the study as an expression of purpose and personal security, (2) ongoing engagement, as a response to flexible processes, a reassuring study environment, and personal commitment, (3) ongoing engagement, challenged by accumulating (practical and emotional) burdens and a reduced sense of connection to the study, and (4) participant-identified needs for a more supportive study experience.

### Theme 1. Joining the study as an expression of purpose and personal security

Theme 1 and associated sub-themes related to joining the study as an expression of purpose and personal security, along with exemplifying quotations are presented in Table 2. The first sub-theme, “Being helpful” refers to the desire to support research or collaborate with the medical team and to help others who are dealing with a similar condition. Some patients with personal backgrounds in research or medicine or who had participated in other studies in the past said that these experiences made them more open to enrolling in the study. As expressed by some, helping would be beneficial not only for others but for oneself too, as it shifts the patient’s role from someone who is always being taken care of and assisted to someone who can help others, enhancing a sense of purpose.

**Table 2:** Structural category: Motivation to participate - Theme: Joining the study as an expression of purpose and personal security

Sub-themes	Participants citing the theme	Exemplifying quotes (Participant’s pseudonym, age)	Theoretical Domains Framework (TDF) Domain(s)
Being helpful	18/30	*"The study made me feel useful. It gave me the impression that I could help caregivers and the medical profession. I saw it as a study not for my own benefit, but for others—for the future."* (Martine, 66 years old) *"For me, it's about advancing medicine and science, making progress. I’m involved, and if it helps move things forward, then it needs to be done."* (Sibylle, 41 years old) "*I think it helps. So, yes, to advance science, even if we know that it's not about something that's really curative, but to help with psychological support, among other things, and everything that's comforting and the side effects of the disease."* (Colette, 74 years old)	Social/Professional Role & Identity; Beliefs about Consequences; Reinforcement
Personal benefit	10/30	*"And also, on some level, you think to yourself, ‘If I take part in a study, maybe—it's selfish, but still—I’ll be monitored a little more closely.’"* (Régine, 70 years old) *"I said yes because if it’s a long-term study, it means I’ll be monitored. I’ll have follow-ups for my cancer, which is a good thing. It reassured me that I wouldn’t be left behind."* (Chantal, 63 years old)	Beliefs about Consequences; Reinforcement; Emotion
Professional/Medical background	6/30	*"I was a pharmacy assistant, so I was already familiar with all the medical procedures."* (Martine, 66 years old) *"Maybe it's my personal background. I'm a psychologist, I'm a guidance counsellor, I've done training, I've done research and so I was open to all these kinds of proposals. It seemed obvious to me that, since I'd asked others to fill in questionnaires for my job, when I was asked to fill in a questionnaire for others, to do the same thing, I thought it would be inappropriate to say no."* (Henriette, 73 years old)	Social/Professional Role & Identity; Skills
Looking beyond Oneself/Transcendence	2/30	*"Not just to be yourself with your own little problems, but to try and go beyond all that… you're part of a community or a collective of women who suffer from more or less the same thing."* (Liliane, 59 years old)	Beliefs about Consequences; Motivation and Goals; Emotion; Reinforcement

The sub-theme “Personal benefit,” was cited as participants’ initial expectation about participation in the study. Being part of a longitudinal study was associated with an expectation to be followed and monitored more closely. This expectation was related to two aspects of patient care: additional medical monitoring (e.g., blood tests) and more frequent meetings with a nurse to whom they could express their concerns and feelings and receive support.

There were also other, less frequently mentioned (lower-level) sub-themes, including “Professional/Medical background” and “Looking beyond Oneself/Transcendence.” The former reflected participants’ prior experience in healthcare or research, which made study participation feel familiar, legitimate, or aligned with their professional values. The latter referred to a desire to contribute to something larger than their personal experience, characterized by a sense of community, solidarity, and the wish to support others facing similar challenges.

### Theme 2. Ongoing engagement, as a response to flexible processes, a reassuring study environment, and personal commitment

This theme and several related sub-themes presented in Table 3.

**Table 3:** Structural category: facilitators of sustained participation - Theme: Ongoing engagement, as a response to flexible processes, a reassuring study environment, and personal commitment

Sub-themes	Participants citing the theme	Exemplifying quotes (participant’s pseudonym, age)	Theoretical domains framework (TDF) domain(s)
No additional cancer center visits/Remote participation	18/30	*"At my center, everything was well organized because my study visit and interview with the study nurse were always scheduled at the same time as my mammography check-up. There was no need to come in just for the study—it was always linked to another appointment."* (Dominique, 73 years old) "[I prefer filling out the questionnaire] *at home. You're more relaxed, you can take your time, go back to it, and think things through. Sometimes, it's not easy to complete it on paper right away—you need to understand it, reread it, and reflect before answering."* (Suzette, 57 years old) *"The appointments were timed to coincide with my follow-up appointments. So in the end, whether I went for a standard follow-up or whether I added 1 h. The appointments [for CANTO] were either before or after, but on the same day [than the follow-up], so either way I had to go."* (Clothilde, 42 years old)	Environmental Context & Resources; Skills
Relationship with study nurse	16/30	*"For me, everything went really well. The nurse was great—we talked about everything that had been done. From the start, it was a positive experience. She was comfortable and made others feel at ease, which I really appreciated."* (Gislhaine, 63 years old) *"What really motivated me was seeing someone. The nurse and I got along well, and I was happy to see someone. That made me even more committed to continuing because I knew I had someone to talk to*—*someone who could answer my questions and understand what was going on."* (Diane, 53 years old)	Social Influences; Emotion; Social/Professional Role & Identity
Increased awareness of side effects	6/30	*"The study opened my eyes to certain things. Filling out the questionnaire made me reflect on the pain I was feeling and other related conditions. […] It helped me realize these were linked to my breast cancer, which led me to ask my doctor questions. […] The regular questionnaires allowed me to track my symptoms and health concerns over time, helping me look for solutions. […] Without this support, I might have just thought, ‘Well, I’m getting older,’ without understanding that these issues were actually linked to my cancer."* (Lisa, 53 years old) *"It actually helped me to tell myself that I wasn't in the process of going completely gaga. I was just actually… because the doctor who proposed us to do it explained to me that when I saw that I couldn't do it any more: 'But that's normal, you're physically and psychologically tired as a result' and he explained to me that my memory was short, so he made me aware of what fatigue and the side-effects of treatment could cause. And that was good, it was reassuring. I found it very reassuring."* (Sarah, 51 years old)	Knowledge; Beliefs about Consequences; Memory, Attention & Decision Processes
Responsibility to commit	12/30	*"We’re committed people by nature. When we make a commitment, we see it through to the end. If we can complete one study and then help with another, we do it. That’s how I see it."* (Colette, 74 years old) *"You know it takes time, you know it's going to take up your time, but if you decide to participate, you do it. I have that kind of principle."* (Sarah, 51 years old)	Social/Professional Role & Identity; Goals
Breaking social isolation	3/30	*"During an illness you're very well looked after at the Center… but you become isolated at the social level. Through the study, I've seen the results of the survey. I've met people who have the same problems as us, and that breaks the isolation."* (Martine, 66 years old)	Social Influences; Emotion
Feeling part of a community	3/30	*"You're part of a community or a collective of women who suffer from more or less the same thing, and to feel that you're part of something bigger than yourself, that helps."* (Liliane, 59 years old)	Social Influences; Emotion
Personal tracking strategy	3/30	*"I kept a personal diary in which I wrote down the particular events that would be noted in the document [for the longitudinal study]."* (Dominique, 73 years old)	Skills

The first sub-theme, “No additional cancer center visits/Remote participation” refers to patients’ preference that study activities coincided with their routine medical visits, rather than requiring separate appointments. Patients appreciated that they were not asked to come to the hospital specifically for the study (i.e., the research visits calendar matched the standard clinical follow-up clinical visit calendar), which simplified their participation, and that they had the option to complete questionnaire surveys for the study from home within a prespecified time window.

The second sub-theme, “Relationship with study nurse,” highlighted the importance participants placed on the presence of a study contact person, and, in particular, the high value accorded to their bond with the study nurse. This relationship was significant for two main reasons: (i) the study nurse represented a “sympathetic ear,” someone who hears them out, and (ii) the nurse served as a reference person for the study, ready to answer all their questions and, in some cases, assisting with the completion of study questionnaires.

The third sub-theme, “Increased awareness of side effects” represents patient narratives discussing how by responding to the same structured questions about their health status at different times during their cancer journey (in the context of the longitudinal study) allowed them to notice the evolution of their side effects. This was especially encouraging and motivating for those who observed reduced side effects. Moreover, questionnaires made them notice the link between experienced side effects and their cancer history rather than simply attributing them to aging.

In addition, the sub-theme “Responsibility to commit,” was generated. Patients often stated that they were determined to complete the study. These discussions went beyond the study scope and were informed by broader life contexts. This sub-theme reflects some patients' approach to life and their dedication to follow through on their life commitments, including their participation in the study.

There were also other, less frequently mentioned sub-themes, including “Breaking social isolation,” “Feeling part of a community,” and “Personal tracking strategy.” The first reflected the value participants placed on the study as a means to counteract the social isolation often experienced after cancer treatment, by fostering connection and reducing feelings of being alone. The second referred to the sense of belonging and solidarity participants felt when engaging with others who had similar experiences. The third captured how some women used the study as an opportunity to systematically monitor their own care trajectories, relying on personal notes or diaries that facilitated their participation over time.

### Theme 3. Ongoing engagement, challenged by accumulating (practical and emotional) burdens and a reduced sense of connection to the study

We summarized patient narratives representing this theme and five associated sub-themes in Table 4.

**Table 4:** Structural category: Challenges to sustained participation - Theme: Ongoing engagement, challenged by accumulating (practical and emotional) burdens and a reduced sense of connection to the study

Sub-themes	Participants citing the theme	Exemplifying quotes (Participant’s pseudonym, age)	Theoretical domains framework (TDF) domain(s)
Lack of feedback and results	17/30	*"I was a bit frustrated not knowing whether the study was already having an impact. After one year, two years, three years, we keep filling things in, but we don’t know what happens to them."* (Régine, 70 years old) *"We haven’t received any feedback, which is a shame. We don’t know what the results will be. There hasn’t been any assessment or analysis. […] I filled in the questionnaire, but I have no idea what the results are. […] On the downside, I have to say it bothers me a little not to have received any results."* (Sarah, 51 years old) *"I'd really like to see the results of this CANTO study, I've always wondered about that."* (Silvia, 51 years old)	Reinforcement; Beliefs about Consequences; Social Influences
Travel burden	17/30	*"Going back to work was a bit of a challenge for me. You still have to juggle your working hours and find time for the study."* (Gishlaine, 63 years old) *"I've had times when I've had little problems and I was thinking: 'Oh, what if I can't drive or if I've got my consultation coming up and I can't go?'."* (Agnès, 75 years old)	Environmental Context & Resources
Participation as reminder of illness	14/30	*"I think we always try to look ahead, to the ‘after,’ believing things will get better. But in the end, the study and its questionnaires bring you back to the present, to moments when things aren’t going well. […] Once you’re no longer in treatment, it’s hard to maintain that connection—even if it’s just a study—because it’s a constant reminder of the illness. […] Every three months, the study reminded me: ‘Oh right, I had cancer, that’s true…’"* (Aude, 51 years old) *"You have to recognize that every time you go back to your notebook to write things down, you're back to being ill."* (Régine, 70 years old) "*What can possibly be an obstacle is thinking: 'Oh yes, I'm still ill because people are still interested in me'. […] you're in this CANTO process thinking: 'Oh yes, I'm being monitored because I'm still considered a person at risk'."* (Arlette, 73 years old)	Emotion; Beliefs about Consequences
Communication difficulties across time points	15/30	*"When you're told you have cancer and then asked to take part in a study, you're lost—you don’t know where you’re going. Maybe it was explained to me, but at first, I didn’t understand. […] The diagnosis threw me off balance. Later on, yes, I learned, and it was explained to me again."* (Corinne, 63 years old) *"I didn’t have a clear schedule. […] With chemotherapy, I had a timeline—I knew my next session was in three weeks in thos dates. […] But with this study, suddenly, I’d receive a questionnaire by email. […] I answered it, but I had no idea if there would be another one in six months, a year, or two years."* (Catherine, 52 years old) *"I didn’t even know if I was still part of the study. Then in May, they told me they were taking a blood sample for it, and I thought,‘Oh dear, I’m still being monitored!’ That’s how I found out."* (Chantal, 63 years old)	Knowledge; Memory, Attention & Decision Processes; Environmental Context & Resources
Repetitiveness of questionnaires	13/30	*"Meeting the deadlines for the study notebooks felt like a burden. It seemed like they kept coming up, and I had to do the same thing over and over again. The questions never really changed—they were always the same."* (Henriette, 73 years old) *"After a while, it became frustrating because I kept thinking, ‘I’m always writing the same thing, filling in the same answers.’ I didn’t notice any improvement* [of her symptoms]*, so it felt a bit tedious."* (Agnès, 75 years old)	Environmental Context & Resources; Memory, Attention & Decision Processes
Staff changes/Lack of continuity	5/30	*"I didn't have the same nurse every time, and I think that can be a barrier."* (Liliane, 59 years old) *"It's been a little while since the nurse retired and said to me: 'it won't be me any more and I'm retiring'. And since then I haven't had any further physical contact. Maybe it was an email or something, but I haven't had any physical contact since she retired."* (Chantal, 63 years old)	Environmental Context and Resources; Social Influences; Emotion;
COVID-19 disrupt	4/30	*"What I found difficult was the COVID. There was this period of COVID when we didn't have face-to-face consultations, it was done by telephone. We often brought the booklet to fill in and gave you another one. In this case, there was no booklet… I found myself with my booklet full, thinking: ‘What do I do with it?’."* (Régine, 70 years old)	Environmental context and resources

The first sub-theme, “Lack of feedback and results” represents a complaint expressed by many patients regarding not receiving updates on the study. Participants emphasized that receiving such updates was important to them, as it confirmed that their contribution was valuable and meaningful.

The second sub-theme, “Travel burden” covers the financial burden related to reaching the center and the time it takes to travel. Moreover, particularly for workers, it was challenging to find time to get to the center.

Regarding the third sub-theme, “Participation as reminder of illness,” some patients expressed that repeatedly reporting their persistent or increasingly prominent side effects made it more difficult to maintain a positive mindset. For participants whose side effects were no longer an issue, the wish to forget about cancer and “move on” with their life was also hindered by their ongoing participation in the study. It is important to note that a few participants voiced this concern as a hypothesis about other patients rather than reflecting their own personal experiences.

The fourth sub-theme, “Communication difficulties accross time points” related to study recruitment, enrollment, conduct, and completion. During enrollment, patients remembered being newly diagnosed, and the invitation to the study sometimes felt unexpected and overwhelming, hindering their comprehension of study-related information. During the study itself, some participants were unsure of the correct study time points, and they also sometimes confused the longitudinal study with other studies that they were participating in. Finally, for some patients, it was unclear whether or not their participation in the study was over.

The final sub-theme, “Repetitiveness of questionnaires” represents the participant burden created by answering the same questions multiple times.

There were also other, less frequently mentioned themes, including “Staff changes/Lack of continuity” and “COVID-19 disrupt.” The first reflected participants’ difficulties in engaging consistently when clinical staff changed frequently, limiting the ability to build rapport and creating uncertainty about follow-up. The second captured how the pandemic disrupted usual procedures, such as face-to-face visits or the exchange of study materials, leading to confusion about expectations and interruptions in participation.

### Theme 4. Participant-identified needs for a more supportive study experience

This theme hihglights perspectives/suggestions for amelioration and is presented along with four associated sub-themes in Table 5.

**Table 5:** Structural category: Suggestions for future improvement - Theme: Participant-identified needs for a more supportive study experience

Sub-themes	Participants citing the theme	Exemplifying quotes (Participant’s pseudonym, age)	Theoretical domains framework (TDF) Domain(s))
Need for choice between digital and paper questionnaires	18/30	*"I think you need to keep both because relying only on digital is risky. The ideal solution is to offer both options and provide access to each."* (Gislhaine, 63 years old) *"I think computer literacy is also a factor. Some age groups may not have had regular exposure to these technologies and could face difficulties using them."* (Dominique, 73 years old)	Skills; Environmental Context & Resources
Need for feedback and study results	15/30	*"I know it's for research and to help others, but beyond that, I don’t know much. If you want to keep people motivated, you need to share results—it’s an incentive. Maybe you could bring participants together […] and thank them, and give them a brief report."* (Suzette, 57 years old) *"We want feedback on how our contributions were used. After all, we all played a part. Something like, ‘Thanks to you, we’ve gained insight into this…’ That’s the kind of acknowledgment we expect."* (Murielle, 56 years old)	Reinforcement; Beliefs about Consequences; Social Influences
Need for emotional or peer support	14/30	*"It’s a shame we never had a group meeting. Sometimes, one person’s memory sparks another’s. Reflection leads to new perspectives. […] Sharing experiences could help others avoid going through the same struggles."* (Martine, 66 years old) *"I would have liked psychological follow-up. I think it's important to talk about yourself, not just your illness. That’s what I feel is missing in healthcare—the psychological support."* (Silvia, 51 years old)	Emotion; Social Influences
Need for reminders	7/30	*"A little text message or email from the study coordinator, saying, ‘How has your last quarter been? Do you have any questions for me?’ could really help when motivation drops. […] A small message like that might be a little push, a reminder that, yes, it's not fun, but it’s important."* (Agnès, 75 years old) *"You forget. I think a reminder—nothing excessive, just a simple nudge—would be helpful, because we do tend to forget."* (Chantal, 63 years old)	Memory, Attention & Decision Processes; Reinforcement; Environmental Context & Resources
Videoconferencing option	4/30	*"Videoconferencing makes a lot of sense because then you have people at the other end who are comfortable with this kind of tool and who don't have to travel."* (Dominique, 73 years old)	Environmental Context & Resources; Skills
Exit interview/Formal closure	4/30	*"I'd find it logical if I had an exit interview, it'll be 6 years so I've handed in my last notebook. I haven't been there for a year, so I'd find it logical to be told that I'm seeing someone from CANTO."* (Régine, 70 years old)	Emotion; Environmental Context & Resources; Social Influences; Beliefs about Consequences

The first sub-theme, “Need for choice between digital and paper questionnaires,” highlights that patients valued having the option to complete questionnaires either on paper or digitally. They suggested that offering this choice in the future would simplify participation, especially for those uncomfortable with one format or the other. Need for adequate navigation of digital instruments was also highlighted, particularly participants voiced this concern for less digitally literate individuals (e.g., elderly, less educated, living in rural areas).

Regarding the second sub-theme, “Need for feedback and study results” patients frequently expressed that, in their opinion, receiving findings from the study would increase their motivation to continue participating and responding to questionnaires. For them, these updates would demonstrate gratitude for their participation and show them that their contributions were valuable.

The third sub-theme, “Need for emotional or peer support” refers to participants’ wish to receive emotional support from other patients and survivors who have had similar experiences and to receive professional psychological support. Patients suggested that it would be useful to create occasions where study participants can gather and interact.

In the final sub-theme, “Need for reminders,” patients expressed concerns about the risk of forgetfulness. They suggested that receiving an additional email reminder, text message, or phone call would help them remember to complete the study questionnaires.

Less frequently mentioned sub-themes included having a “Videoconferencing option” and “Exit interview/Formal closure.” Participants highlighted videoconferencing as a convenient alternative to in-person meetings, reducing travel and potentially improving attendance and motivation, especially following the rise of remote communication during the COVID-19 pandemic. Additionally, many expressed a desire for a formal closure to their participation, such as an exit interview or a final communication, to acknowledge their contribution and provide updates on the study progress.

## Discussion

In this qualitative study, we conducted 17 individual interviews and two focus groups with five and eight participants each, among women who had a history of early-stage breast cancer and had taken part in the longitudinal CANTO cohort. Our objective was to gain a deeper understanding into experiences and perspectives on participation in longitudinal research, with the broader goal of enhancing study procedures and the participant’s experience, and of minimizing attrition. We identified four overarching themes: (1) Joining the study as an expression of purpose and personal security; (2) Ongoing engagement, as a response to flexible processes, a reassuring study environment, and personal commitment; (3) Ongoing engagement, challenged by accumulating (practical and emotional) burdens and a reduced sense of connection to the study; and (4) Participant-identified needs for a more supportive study experience.

Although our primary aim was to explore factors contributing to attrition in cohort research, the first theme relates to participants’ initial motivation to enroll in a longitudinal study. The implemented interview and focus group guides indeed intentionally included prompts about participants’ reasons for joining CANTO. This decision reflected evidence that early expectations and motivations can shape later engagement, influencing both sustained participation and eventual withdrawal [29]. Participants often linked their enrollment motivations with the factors that affected their long-term involvement, reinforcing the value of examining these elements together within the same qualitative inquiry.

First and foremost, our findings highlight the importance of clear and consistent communication with participants throughout a longitudinal study, beginning with a clear explanation of the study’s rationale and purpose at enrollment, then reiterating this information during follow-up. Sub-themes related to communication (e.g., “Relationship with study nurse”; “Lack of feedback and results”; “Communication difficulties across time points”) were central across all phases of participation, therefore influencing initial enrollment as well as ongoing engagement and retention. Given the frequent gaps in patient understanding after informed consent, clear and thorough communication from the outset is essential to support informed participation and long-term engagement [30]. Patients expressed a strong desire to be clearly informed about the study aims, participation expectations, and the value of their contribution. This transparency would foster a true shift toward a more conscious sense of participation [31, 32] and promote empowerment. [33]

Improved communication can also help prevent misconceptions about the benefits of participating in an observational longitudinal study. Several CANTO participants expected personal benefit with enrollment, particularly closer monitoring/exams or additional support during their cancer journey. While such expectations may boost early engagement, they can harm long-term retention when the study does not provide enhanced clinical follow-up. As an observational cohort, neither CANTO offers specialized care nor it allows to adjust clinical management based on symptom or quality of life data. When participants perceive their involvement as additional surveillance or expect healthcare professionals to act on study data, and these expectations are not met, this gap can lead to disappointment, disengagement, and attrition once it becomes clear that study procedures are separate from routine care [34, 35]. All ambiguities regarding the distinctions between routine medical care and procedures specific to the longitudinal study should be therefore promptly clarified.

Communication and information about the study should also be provided at the appropriate time. Some patients may feel overwhelmed if invited to enroll in a longitudinal study on the same day as their cancer diagnosis. It is important to respect the emotional sensitivity of this period while adhering to ethical standards and balancing study requirements (for example, CANTO mandates a baseline pre-treatment visit that must occur before any cancer treatment), aiming to minimize patient burden and support informed consent [36, 37].

Providing clear information about the scope of data collected and the characteristics of the instruments used can also help alleviate the feeling of “repetitiveness” associated with questionnaires. A key strategy is to clearly communicate the study goals and explain why collecting patient-reported information through these instruments at multiple time points (often long after initial diagnosis) is essential, emphasizing that seemingly similar questions may capture distinct, sensitive, and important health data [38, 39].

At the final stage of the longitudinal study, some participants reported uncertainty about whether the study was still ongoing or if their participation had concluded, highlighting the need for clear communication until study completion. Providing formal closure may help address this issue. Overall, study completion information along with all the aforementioned communications about the study can be effectively delivered via different channels, including during in-person appointments with study personnel (e.g., study nurse) or via telephone or email [40, 41], information leaflets and printed material, or posts and newsletter in a digital format [42].

This qualitative analysis also identified facilitators of participation in longitudinal research, including minimizing the burden of additional travel to the cancer center. Patients reported that scheduling study visits on the same day as medical appointments and having the option to complete self-reported instruments remotely—an approach previously associated with improved retention in clinical trials [43]—simplified their participation. When asked about additional facilitators, participants also valued the opportunity to reflect on their symptoms and functional impact when completing self-evaluation questionnaires, underscoring the importance of these instruments for improved detection and management of health-related issues [38].

On the contrary, lack of feedback on how data collected in CANTO were used and what research outcomes resulted from the exploitation of such data was a key factor challenging sustained participation in our analysis. Participants highlighted a need for regular updates on study progress and results, alongside concerns about understanding the purpose and scope of extensive, long-term questionnaire-based data collection. To address this need, patient engagement might be strengthened by sharing study findings [44] through diverse channels such in-person seminars, newsletters, online webinars, but also via innovative, patient-friendly formats like podcasts and video summaries designed for a lay audience [45].

It is particularly clear from our analysis that repetitive questionnaires pose a major challenge to sustained participation. Study instruments should be carefully selected to address specific research questions, with attention to readability, clarity, and acceptability to accommodate diverse patient backgrounds [46]. Reducing the number and redundancy of questionnaires can help minimize burden. Additionally, collaboration between healthcare providers and researchers to jointly collect data for clinical and research purposes may further reduce patient fatigue related to study participation [12]. Furthermore, the next generation of patient-generated data should aim at collecting innovative data sources beyond traditional patient-reported outcomes and patient-reported experience questionnaires, including, for example, novel activity data from wearable devices, maximizing passive data collection and reducing burden on participants [47].

We believe that interpreting the findings of the present study in light of the TDF provides additional conceptual clarity regarding the behavioral processes that shape survivors’ sustained participation in a longitudinal cohort [48, 49]. Several of the identified sub-themes align with specific TDF domains [50, 51], including: social/professional role & identity (e.g., “Being helpful”), environmental context and resources (e.g., “No additional cancer center visits/remote participation”; “Travel burden”), social influences (e.g., “Relationship with study nurse”), beliefs about consequences (e.g., “Personal benefit”), reinforcement (e.g., “Lack of feedback and results”), emotion (e.g., “Participation as a reminder of cancer”; “Need for emotional or peer support”), knowledge (e.g., “Increased awareness of side effects”), memory, attention and decision processes (e.g., “Need for reminders”), and skills (“Need for choice between digital and paper questionnaires”). Interpreting the results through this framework clarifies how practical constraints, interpersonal interactions, and emotional responses intersect to influence long-term engagement. This theoretical interpretation strengthens the applicability of the findings by highlighting behavioral determinants that may be targeted to reduce attrition, such as improving communication about study progress, enhancing navigation support, optimizing digital accessibility, and reinforcing the perceived value of continued participation [52].

Turning to strategies to reduce attrition, a systematic review of 28 population-based cohort studies found that incentives consistently improved retention, with higher-value incentives being more effective [41]. Reminder methods, repeat questionnaires, and alternative data collection modes also had varying positive effects. Flexible data collection particularly benefited face-to-face studies, though overall evaluation of retention strategies was often unsystematic. A meta-analysis of 143 longitudinal cohorts by Teague et al. reported a mean retention rate of 73.5% (Standard Deviation [SD] = 20.1%), over 4.3 years (SD = 5.0) and outlined that barrier reduction strategies—such as flexible data collection and remote questionnaires— during the follow-up of longitudinal studies led to approximately 10% reduction in attrition [8]. These participant-centered, burden-reduction strategies improved retention more effectively and sustainably than frequent reminders. Emerging digital strategies show promise but have a risk to widen the digital divide across age, socioeconomic, and geographic groups, highlighting the need for tailored digital support for those most in need [53]. Some participants in our analysis also voiced concern for others facing these barriers. Overall, costs of these retention strategies vary widely, with some intensive strategies being expensive yet offering uncertain benefits [54]. Modest incentives (when permitted by law) can be useful, but data on their cost-effectiveness remain limited.

Our study presents several limitations. First, we reached out to a group of patients that were or had been enrolled on a longitudinal cohort (CANTO) and that were still participating in the study at the time of these analyses or finished regular follow-up, whereas we could not include patients that had dropped out of the cohort. Being unable to contact or collect feedback from participants who withdrew consent and discontinued participation in CANTO restricts our understanding of their perspectives. The impact on results include possible underestimation of challenges faced by dropouts, and findings that may overrepresent engaged, motivated participants. Future research should prioritize assessing the dropped-out subgroup to better elucidate reasons for discontinuation, although this might be challenging from a regulatory standpoint. Second, participation was done on a voluntary basis. Therefore, our sample was already pre-selected for highly motivated patients, with possible underreporting of barriers or negative experiences. Third, the generalizability of our results may also have been impacted by the characteristics of our sample. For example, despite the provision of technical support and practice sessions, the requirement for virtual participation may have introduced selection bias favoring individuals with higher digital literacy. We also acknowledge that cultural differences may influence patient experiences and preferences in longitudinal research participation. Factors such as communication styles, health beliefs, trust in research, and expectations of care can vary across cultural contexts and potentially impact motivation, engagement, and retention. While our study was conducted within a specific population (i.e., women with a history of breast cancer), exploring cultural, social, and environmental influences in diverse settings is an important area to ensure study designs and communication strategies are appropriately tailored and inclusive. Importantly, existing research on retention strategies in other cancer populations, such as men with prostate cancer, reveals consistent findings regarding common facilitators and barriers to participation, including logistical challenges, perceived burden, and the need for clear communication and support [55]. This suggests that many retention factors may be broadly applicable across cancer cohorts, reinforcing the potential for generalizable patient-centered strategies to improve longitudinal study retention. Finally, the representativity of the sample in terms of ethnicity could not be assessed due to current regulations preventing collection of these type of data.

Strengths lie in the use of qualitative methods, which capture rich, nuanced understanding of survivors’ experiences and perceptions. Drawing participants from the well-established CANTO longitudinal cohort ensured relevance and engagement, while the application of a theoretical framework provided additional clarity in interpreting behavioral factors influencing retention. The inclusion of a quite diverse sample across age and backgrounds enhanced the transferability of findings within similar clinical populations. Importantly, the study directly integrated patient perspectives, yielding actionable insights to inform the design of more patient-centered longitudinal research.

In conclusion, findings of this qualitative analysis will inform strategies to reduce attrition and enhance the overall participant experience in longitudinal studies. An example of such an intervention will be piloted within the CANTO cohort (CANTO-ATTITUDE nested randomized trial), powered by a digitally-enabled insfrastructure [56]. Future research should also investigate reasons for non-participation in longitudinal cohorts, encompassing both patients who decline initial enrollment and those who withdraw during follow-up. Gaining insight into their motivations, challenges, and perspectives is essential for developing more patient-centered protocols, enhancing recruitment strategies, and minimizing attrition.

## Supplementary Information

Below is the link to the electronic supplementary material.

(PDF 442 kb)

(DOCX 38 kb)

(PPTX 76 kb)

## Data Availability

The datasets generated during and/or analysed during the current study are are available from the corresponding author on reasonable request.
