# Supplementary material for: ATTITUDE - Addressing attrition in longitudinal cancer cohorts: an in-depth qualitative analysis of experiences and perspectives on participation in longitudinal studies among breast cancer survivors
Source: Breast Cancer Res Treat. 2026 Feb 11;216(1):4. doi: 10.1007/s10549-026-07904-w (PMC12891045; doi:10.1007/s10549-026-07904-w)
Supplement: Supplementary file 2 — (DOCX 38 kb) [file 10549_2026_7904_MOESM2_ESM.docx]

**APPENDIX II**

**“ATTITUDE - Addressing Attrition in Longitudinal Cancer Cohorts: An In-Depth Qualitative Analysis of Experiences and Perspectives on Participation in Longitudinal Studies Among Breast Cancer Survivors”**

**Contents**

[**Interview Guide** 2](#_Toc216454580)

[**Focus Group guide** 4](#_Toc216454581)

[**Socio-Demographic Questionnaire** 5](#_Toc216454582)

## **Interview Guide**

**Requesting consent for recording the session for subsequent thematic analysis.**

**1. Introduction: Presentation, brief overview of the study and explanation of the interview procedure.**

**2. Experience in a Longitudinal Study:**

- How long have you been involved in the study?
- Can you describe your experience with the study, particularly regarding data collection, including completing questionnaires?
- Did you find it easy to complete the questionnaires?
- Did you use the paper or electronic version of the questionnaire?
- Did you find the length of the questionnaires and the time required to complete them appropriate?
- Was the frequency of completing the questionnaires suitable?
- What did you think of the content of the questionnaires? Was it clear and appropriate? Were you able to ask questions if anything was unclear?
- Did you find any questionnaire particularly difficult to complete?

**3. Motivation to Participate in a Longitudinal Study:**

- What motivated you to participate in the study?

**4. Facilitators and Challenges to Participation:**

- What factors helped or hindered your participation in the study?
- What might cause you to stop participating in this type of study?

**5. Information about the Longitudinal Study:**

- Do you feel that you were well informed before starting the study? Were the explanations you received clear and sufficient?

**6. Opinion on Digital Tools:**

- Do you prefer digital or paper questionnaires?
- Would you prefer to complete the questionnaires remotely (at home) or at the hospital?
- Would you be open to participating in a fully digital survey?
- Can you think of any barriers to using digital tools, either for yourself or others?

**7. Opinion on the End of the Study:**

- If you have completed your participation in the study, how would you describe your experience with the conclusion of the study?

**8. Facilitators to Participation in a Longitudinal Study:**

- What can be done to make it easier to participate in the study?
- How can we help keep you motivated during the study?
- How can we assist you in traveling to the hospital for study participation?
- How can we make it easier for you to complete the study questionnaires?
- How can we express our appreciation for your participation?

**9. Do you have anything else to add? Is there anything you'd like to revisit?**

**Thank you for your participation**

## **Focus Group guide**

**Requesting consent for recording the session for subsequent thematic analysis.**

**Introduction: Presentation, brief overview of the study and explanation of the focus group procedure.**

- **Housekeeping ad Overview**: brief presentation of the researcher/observer, provision of general information on how the focus group will run.
- **Participant Introductions**: brief introduction of each participants
- **Introduction to the Attitude Project**: brief review of the study

**Participants interacations and discussion**

**Topics prompted:**

**a. What was your experience in a longitudinal study?**

Follow-up questions (if needed)

• Could you describe your overall experience participating in the study, including your interactions with the study team, the hospital visits, and the questionnaires?

**b. What were your motivation to participate in a longitudinal study?**

Follow-up questions (if needed)

• If you had to explain to another patient why you chose to participate, keeping only the core of your personal motivation, what would you tell them?

**c. What were the facilitators and challenges to you participation in a longitudinal study?**

Follow-up questions (if needed)

• Could you describe the reasons that either supported or hindered your ongoing participation, and the factors that could have led to your withdrawal?

**d. What are your opinion on emerging technology including digital tools?**

Follow-up questions (if needed)

• Considering both convenience and accessibility, which questionnaire format (paper or fully digital) do you prefer, and would you be willing to participate in a research study implemented using exclusively digital tools?

**Final thoughts and revisiting topics**

**Thank participants**

**Outline of the next steps**

## **Socio-Demographic Questionnaire**

- How old are you?
- What is your highest level of education?
- What is your current occupation?
- What is your family situation?
- Do you have any children?
- Where do you live? (Large city [over 20,000 inhabitants], Small city [under 20,000 inhabitants], Village)
- How far is your home from the cancer care facility? (In the same town, Less than 50 km, 50-150 km, More than 150 km)
- Which comprehensive cancer center did you receive treatment at?

**Thank you for your participation**
