# Supplementary figures and images for: ATTITUDE - Addressing attrition in longitudinal cancer cohorts: an in-depth qualitative analysis of experiences and perspectives on participation in longitudinal studies among breast cancer survivors
Source: Breast Cancer Res Treat. 2026 Feb 11;216(1):4. doi: 10.1007/s10549-026-07904-w (PMC12891045; doi:10.1007/s10549-026-07904-w)

## Slide 1
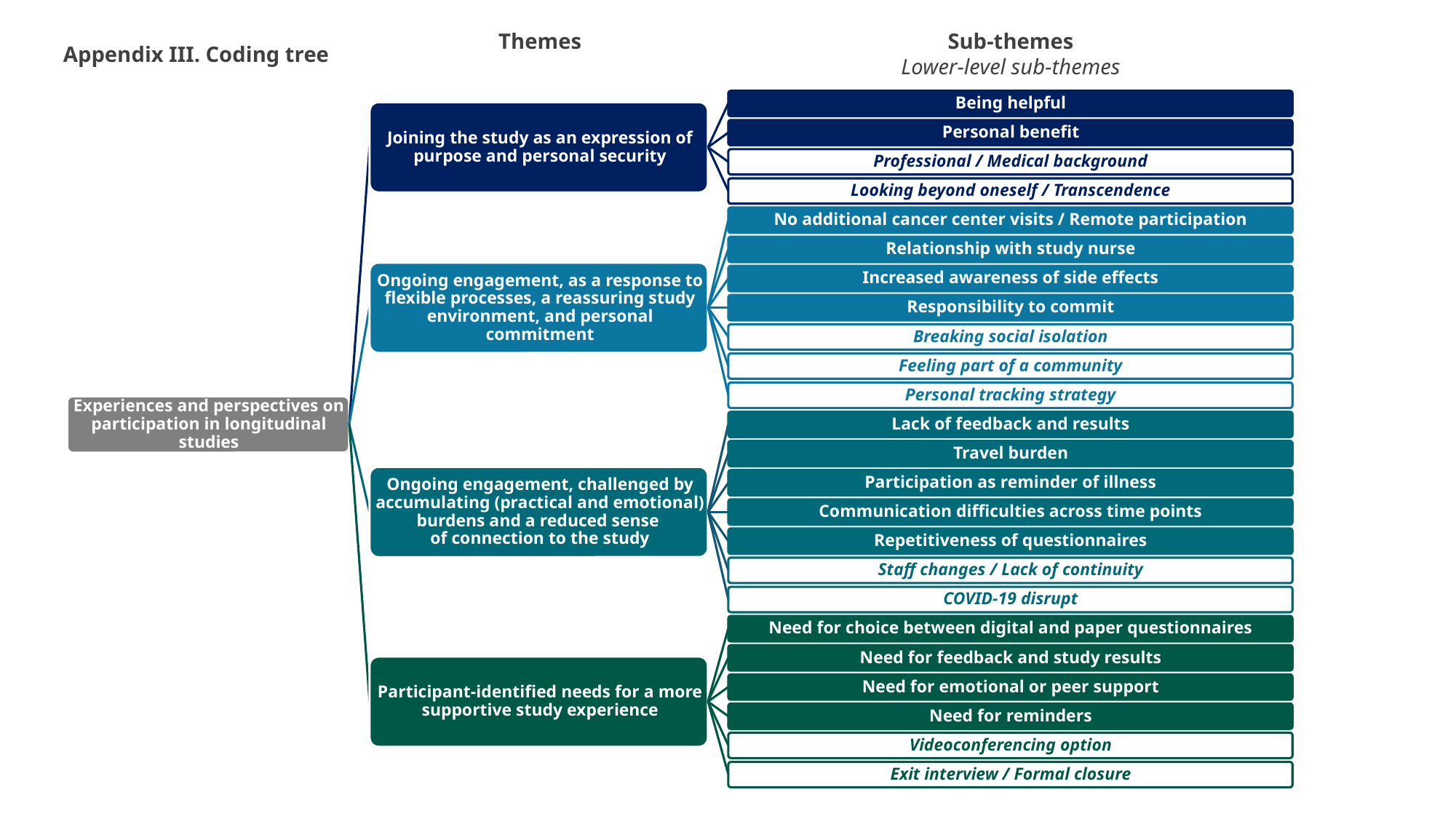

Themes
Sub-themes
Lower-level sub-themes
Appendix III. Coding tree

Supplement: Supplementary file 3 — (PPTX 76 kb) [file 10549_2026_7904_MOESM3_ESM.pptx]
